# Discontinuation of afterload-reducing drugs decreases left ventricular outflow tract obstruction in hypertrophic obstructive cardiomyopathy

**DOI:** 10.3389/fcvm.2024.1403422

**Published:** 2024-07-16

**Authors:** Anselm A. Derda, Malin Abelmann, Kristina Sonnenschein, Jan-Thorben Sieweke, Udo Bavendiek, Johann Bauersachs, Thomas Thum, Dominik Berliner

**Affiliations:** ^1^ Department of Cardiology and Angiology, Hannover Medical School, Hannover, Germany; ^2^ Institute of Molecular and Translational Therapeutic Strategies (IMTTS), Hannover Medical School, Hannover, Germany

**Keywords:** HCM, pharmacotherapy, LVOT, cardiomyopathy, heart failure, mavacamten

## Abstract

**Background:**

Hypertrophic cardiomyopathy (HCM), the most common genetic heart disease, is classified into hypertrophic non-obstructive and hypertrophic obstructive cardiomyopathy (HOCM). Patients with HOCM and coexisting heart failure or arterial hypertension are often prescribed afterload-reducing drugs. Although recommended in current guidelines, data on the direct effect of discontinuing afterload-reducing medication are scarce. This study aims to demonstrate the benefit of discontinuing afterload-reducing medication in HOCM patients.

**Methods:**

This monocentric retrospective analysis included 24 patients with HOCM with afterload-reducing medication, including angiotensin-converting enzyme inhibitors, angiotensin-1 receptor blocker and dihydropyridine-calcium channel blocker, at their first outpatient visit. Effects of discontinuing this medication on LVOTO were examined compared to patients with persistent use despite medical advice.

**Results:**

16 patients discontinued their afterload-reducing drugs, resulting in a significant decrease in median LVOT gradient from 86.5 [60.5–109.3] mmHg to 61.5 [28.3–97.50] mmHg (*p* = 0.0004). In 6 patients, beta-blocker therapy was initiated simultaneously, or the dose was increased. Regardless, LVOT gradient reduction was also significant in the remaining 10 patients (*p* = 0.001). The gradient was not changed significantly in the 8 patients continuing their afterload-reducing medication.

**Conclusions:**

Discontinuation of afterload-reducing drugs significantly decreases LVOTO. Our study underscores the significance of abstaining from afterload-reducing drugs in HOCM patients, particularly in patients with concomitant hypertension or heart failure. According to recently published European guidelines, HOCM patients should preferably be treated with beta-blockers or non-dihydropyridine-calcium channel blockers.

## Introduction

Hypertrophic cardiomyopathy (HCM) is the most common genetic heart disease, with a prevalence of up to 1 in 200–500 (1). Pathologic growth of cardiomyocytes, often asymmetrically affecting the interventricular septum, can lead to a narrowing of the left ventricular outflow tract (LVOT) and thus may provoke left ventricular outflow tract obstruction (LVOTO) (2). Depending on the presence of LVOTO, HCM is classified into hypertrophic non-obstructive cardiomyopathy (HNCM) and hypertrophic obstructive cardiomyopathy (HOCM), with the latter accounting for 70% of cases (3). Outflow tract obstruction, defined as a gradient ≥30 mmHg either at rest or on physiological provocation (4), is often associated with more severe symptoms of dyspnea, dizziness, syncope, fatigue, and an increased risk of sudden cardiac death (SCD) compared with the non-obstructive form (5). When addressing the management of HOCM through pharmacological interventions, it is crucial to consider several important aspects, especially in patients with concomitant arterial hypertension (AH) or heart failure (HF). Current guidelines from the European Society of Cardiology (ESC) and the American College of Cardiology (ACC) recommend anti-obstructive therapy, i.e., beta-blockers or verapamil to reduce gradient and symptoms (6–8). In addition, afterload-reducing drugs, including angiotensin-converting enzyme inhibitors (ACEis), angiotensin receptor blockers (ARBs) and dihydropyridine calcium channel blockers (CCB-DHPs), are not recommended because they are suspected of worsening the obstruction and symptoms (6, 7). The coexistence of AH in a significant proportion of HOCM patients often leads to inadvertent administration of afterload-reducing drugs, worsening the LVOTO. Furthermore, ACEis and ARBs have convincingly been shown to significantly improve adverse remodeling in HF patients (9–11). Among the “fantastic four” medication used to treat heart failure, drugs that lower afterload are an essential component (12). Because these physiologic processes are central to hypertrophic cardiomyopathy (HCM), there is a tendency to prescribe these agents for their potential benefit, inadvertently worsening the LVOTO and symptoms of discomfort. Therefore, this study aims to demonstrate the positive clinical effect of guideline-compliant discontinuation of afterload-reducing medication in HOCM patients in a real-world scenario.

## Methods

This monocentric retrospective study was conducted following the ethical principles of the Declaration of Helsinki (13). Written informed consent was obtained from each patient. The study was approved by the ethics committee of the MHH (Ethics vote no. 5632).

The study took place at the special outpatient clinic for cardiomyopathies of the Department of Cardiology and Angiology at Hannover Medical School (MHH). The HCM registry was examined to identify patients with HOCM being on afterload-reducing drugs at their first outpatient visit and returning to MHH for routine cardiological follow-up. ACEis, ARBs and CCB-DHPs were classified as afterload-reducing drugs. No patient was on angiotensin receptor neprilysin inhibitor (ARNI) at initial presentation. Therefore, use of ARNI has not been considered in this analysis.

The diagnosis of HCM was based on the latest ESC guidelines for the diagnosis and management of hypertrophic cardiomyopathies: patients with familial or genetically diagnosed HCM with wall thickness ≥13 mm in one or more left ventricular myocardial segments or patients with wall thickness ≥15 mm in the absence of any other cause of hypertrophy (7, 8). LVOTO was defined as an instantaneous peak Doppler LVOT pressure gradient ≥30 mmHg at rest or during physiological provocation, such as the Valsalva maneuver or after nitroglycerin administration (7, 8).

### Echocardiography

An echocardiographic examination was performed using Philips ultrasound systems. Echocardiography was performed according to the recommendations of the American Society of Echocardiography and the European Association of Cardiovascular Imaging (14–16). LVOTO was assessed in the apical five-chamber view. A modified Bernoulli equation was used to automatically convert the maximum flow velocity into the LVOT pressure gradient (17). The maximum measurable gradient at rest, during the Valsalva maneuver, after nitroglycerin application or during a combination of these procedures is the maximum LVOT gradient listed below.

Interventricular septal thickness in diastole (IVSd) and left ventricular end diastolic diameter (LVEDD) were assessed in the parasternal long axis during diastole. Left atrial size was evaluated using left atrial parasternal long axis (LA PLAX). The LV mass was calculated using the following parameters: IVSd, LA PLAX and thickness of left ventricular posterior wall in diastole (18). Next, the LV mass-index was calculated by dividing the LV mass by the body surface area.

### Statistical analysis

Categorical variables are presented as numbers and percentages. Continuous variables are reported as mean ± standard deviation (SD) for normally distributed data or as median and interquartile range (IQR) for non-normally distributed data. The normality of continuous data distribution was tested using the Kolmogorov-Smirnov and Shapiro-Wilk tests. In the event that either test yielded a statistically significant result, the presumption of a non-normal distribution was assumed. Differences between the two outpatient visits were assessed using the Wilcoxon test for paired non-normally distributed data and the *t*-test for paired normally distributed data. The Mann-Whitney *U*-test was utilized for unpaired and non-normally distributed data, and the *t*-test for unpaired and normally distributed data. The distribution of unpaired categorical variables was analyzed using Chi-square or Fisher's exact test.

All statistical analyses were performed using the Statistical Package for the Social Science, version 28.0 (IBM SPSS, Armonk, NY, USA) and GraphPad Prism 8.4.3 (GraphPad Inc., La Jolla, CA, USA). Statistical significance was defined by a *p*-value <0.05. Figures were created using GraphPad Prism 8.4.3 (GraphPad Inc., La Jolla, CA, USA).

## Results

A total of 24 HOCM patients with concomitant AH were included in the analysis. The mean age of the patients who discontinued afterload-reducing medication was 68.0 ± 7.5 years at their first visit to the special outpatient clinic. The mean age of patients continuing their afterload-reducing medication was 62.9 ± 7.3 years at their first visit (*p* = 0.11). 16 patients (66.7%) discontinued afterload-reducing medication; the remaining 8 (33.3%) continued taking it against medical advice. The proportion of men in the two groups was 44% and 50% respectively.

The baseline characteristics of our two cohorts, those of patients who discontinued afterload-reducing medication and those of patients who continued afterload-reducing medication, are demonstrated in Table 1. There were no significant differences between the two groups with regard to cardiovascular risk factors such as an increased body mass index (BMI) or diabetes. Conversely, a significant higher proportion of patients who did not stop afterload-reducing medication were smokers (*p* = 0.02). A breakdown of demographic characteristics by gender is provided in Supplementary Table S1. A comparison of how these parameters changed towards the second outpatient visit is reported in Table 2.

**Table 1:** Baseline characteristics of HOCM patients who discontinued afterload-reducing medication (left; *n* = 16) and HOCM patients who continued afterload-reducing medication (right; *n* = 8) at their first outpatient visit at MHH.

** **	First visit (discontinuation)	First visit (continuation)	*p*-value
Demographics			
Age (years)	68.0 ± 7.5	62.9 ± 7.3	0.11
Number of males (%)	7 (43.8)	4 (50.0)	1
BMI (kg/m^2^)	28.7 ± 4.1	31.1 ± 6.6	0.27
Diabetes (%)	1 (6.3)	2 (25.0)	0.25
Smoking (%)	2 (12.5)	6 (75)	0.02
Systolic blood pressure (mmHg)	135.1 ± 18.9	135.4 ± 14.5	0.97
Diastolic blood pressure (mmHg)	74.6 ± 10.4	78.0 ± 7.9	0.45
Arterial hypertension (%)	16 (100)	8 (100)	1
Pharmacotherapy			
Beta-blocker (%)	10 (62.5)	5 (62.5)	1
ACEi (%)	10 (62.5)	7 (87.5)	0.35
ARB (%)	4 (25.0)	0 (0)	0.26
CCB-DHP (%)	6 (37.5)	2 (25.0)	0.67
Non-DHP CCB (%)	2 (12.5)	1 (12.5)	1
MRA (%)	2 (12.5)	0 (0)	0.54
Diuretics (%)	9 (56.3)	5 (62.5)	1
ASA (%)	3 (18.8)	4 (50.0)	0.17
Marcumar (%)	2 (12.5)	1 (12.5)	1
DOAC (%)	4 (25.0)	1 (12.5)	0.63
Statin (%)	8 (50.0)	4 (50.0)	1
Symptoms			
NYHA I or II	12 (75.0)	6 (75)	1
NYHA III or IV	4 (25.0)	2 (25)	1
Palpitations (%)	3 (18.8)	1 (12.5)	1
Syncope (%)	0 (0)	0 (0)	1
Echocardiography			
LVOT gradient max. (mmHg)	86.5 [60.5–109.3]	56.5 [41.5–63.3]	0.02
IVSd (mm)	18.8 ± 2.6	19 ± 3.5	0.88
LA PLAX (mm)	45.6 ± 5.8	39.3 ± 5.5	0.02
LVEDD (mm)	43.0 ± 5.8	39.6 ± 9.4	0.36
LV Mass (g)	266.3 ± 54.6	255.1 ± 120.9	0.76
LV Mass-Index (g/m^2^)	138.0 ± 29.4	125.7 ± 50.9	0.47
Diastolic dysfunction (%)	16 (100)	8 (100)	1
Mitral valve regurgitation at least moderately severe (%)	10 (62.5)	2 (25.0)	0.19
LVEF ≥ 50%	16 (100)	8 (100)	1

HOCM, hypertrophic obstructive cardiomyopathy; BMI; body mass index; ACEi, Angiotensin converting enzyme inhibitor; ARB, angiotensin-1 receptor blocker; CCB-DHP, dihydropyridine calcium channel blocker; non-DHP CCB, non-dihydropyridine calcium channel blocker; MRA, mineralocorticoid receptor antagonist; ASA, acetylsalicylic Acid; DOAC, Direct oral anticoagulants; NYHA, New York Heart Association; LVOT, left ventricular outflow tract; IVSd, interventricular septal thickness in diastole; LA PLAX, left atrial parasternal long axis; LVEDD, left ventricular end diastolic diameter; E/E;LVEF, left ventricular ejection fraction.

**Table 2:** Comparison of parameter differences between second and first outpatient visit between patients HOCM patients who discontinued afterload-reducing medication (left; *n* = 16) and HOCM patients who continued afterload-reducing medication (right; *n* = 8). Delta values were created using data from both outpatient visits and these delta values were then compared with each other.

** **	Discontinuation	Continuation	*p*-value
Demographics			
Δ Time between outpatient visits (days)	192.5 [112.8–339.3]	166.0 [46.5–365.0]	0.52
Δ Systolic blood pressure (mmHg)	7.3 ± 32.3	−14.0 ± 18.8	0.12
Δ Diastolic blood pressure (mmHg)	0.5 [−4.0–12.0]	1.5 [−7.0–10.0]	0.94
Pharmacotherapy			
Δ Beta-blocker (%)	+1 (+16.6)	+1 (+33.3)	1
Δ ACEi (%)	−10 (−100)	0 (0)	<0.001
Δ ARB (%)	−4 (−100)	0 (0)	1
Δ CCB-DHP (%)	−5 (−83.3)	0 (0)	0.11
Δ non-DHP CCB (%)	+2 (+14.3)	0 (0)	1
Δ MRA (%)	+5 (+35.7)	0 (0)	0.12
Δ Diuretics (%)	+1 (+14.3)	0 (0)	1
Symptoms			
Δ NYHA I or II	0 (0)	0 (0)	1
Δ NYHA III or IV	0 (0)	0 (0)	1
Δ Palpitations (%)	−1 (−33.3)	0 (0)	1
Δ Syncope (%)	+1 (+6.3)	0 (0)	1
Echocardiography			
Δ LVOT gradient max. (mmHg)	−31.7 ± 33.8	14.2 ± 23.8	0.0024
Δ IVSd (mm)	1.0 [0.0–1.75]	−1.0 [−2.0–0.8]	0.10
Δ LA PLAX (mm)	−0.8 ± 4.0	1.5 ± 7.5	0.34
Δ LVEDD (mm)	−0.4 ± 8.5	1.9 ± 8.5	0.54
LV Mass (g)	16.0 ± 68.0	17.8 ± 109.2	0.96
LV Mass-Index (g/m2)	5.9 ± 32.1	10.8 ± 52.3	0.78
Δ Mitral valve regurgitation at least moderate (%)	+3 (+50)	+1 (+16.6)	0.55

ACEi, angiotensin converting enzyme inhibitor; ARB, angiotensin-1 receptor blocker; CCB-DHP, dihydropyridine calcium channel blocker; non-DHP CCB, non-dihydropyridine calcium channel blocker; MRA, mineralocorticoid receptor antagonist; NYHA, New York Heart Association; LVOT, left ventricular outflow tract; IVSd, interventricular septal thickness in diastole; LA PLAX, left atrial parasternal long axis; LVEDD, left ventricular end diastolic diameter.

**Figure 1:**
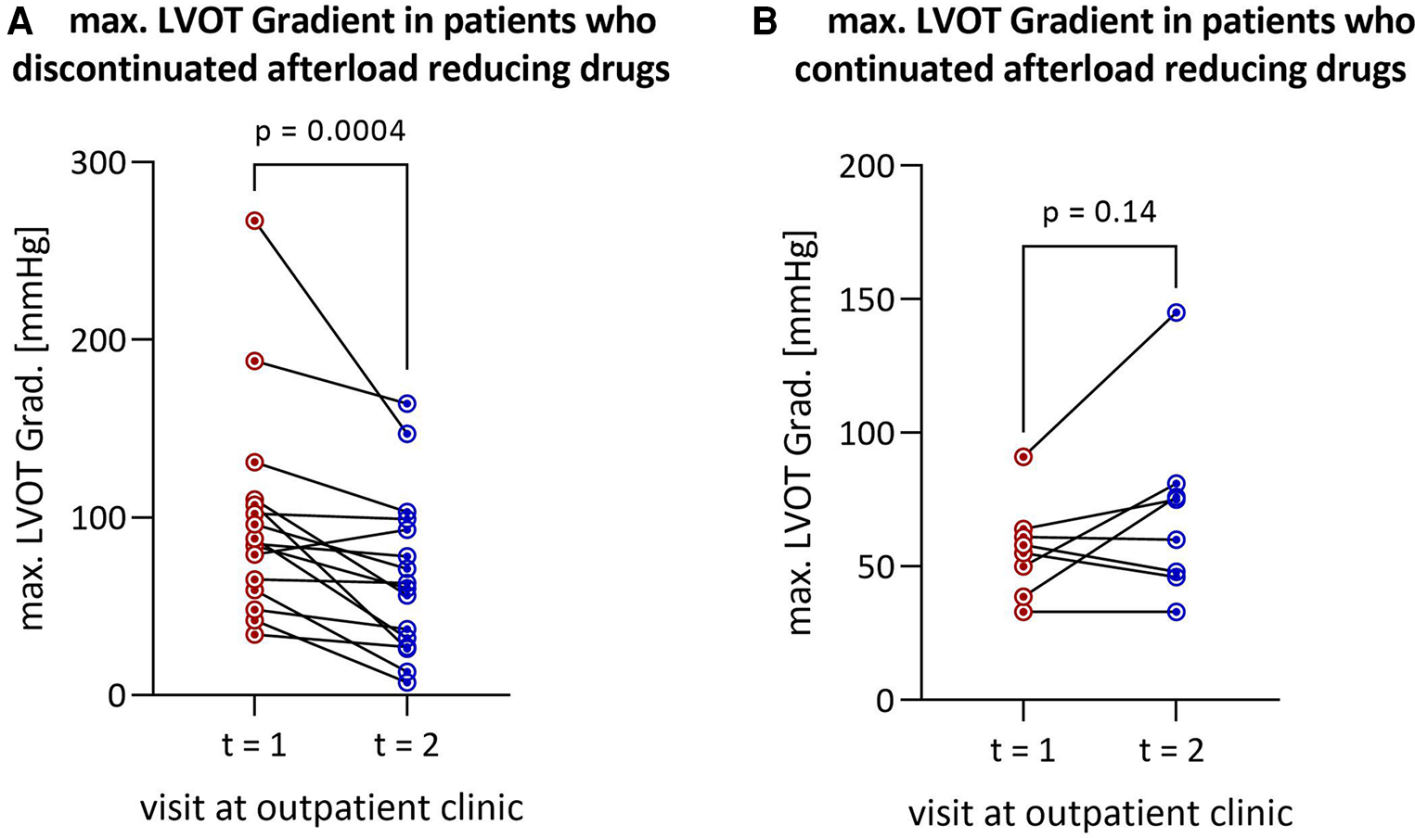
Reduction in LVOT gradient after discontinuation of afterload-reducing drugs in (**A**) all HOCM patients (*n* = 16) and (**B**) patients remaining on afterload-reducing medication (*n* = 8). LVOT, left ventricular outflow tract; HOCM, hypertrophic obstructive cardiomyopathy.

**Figure 2:**
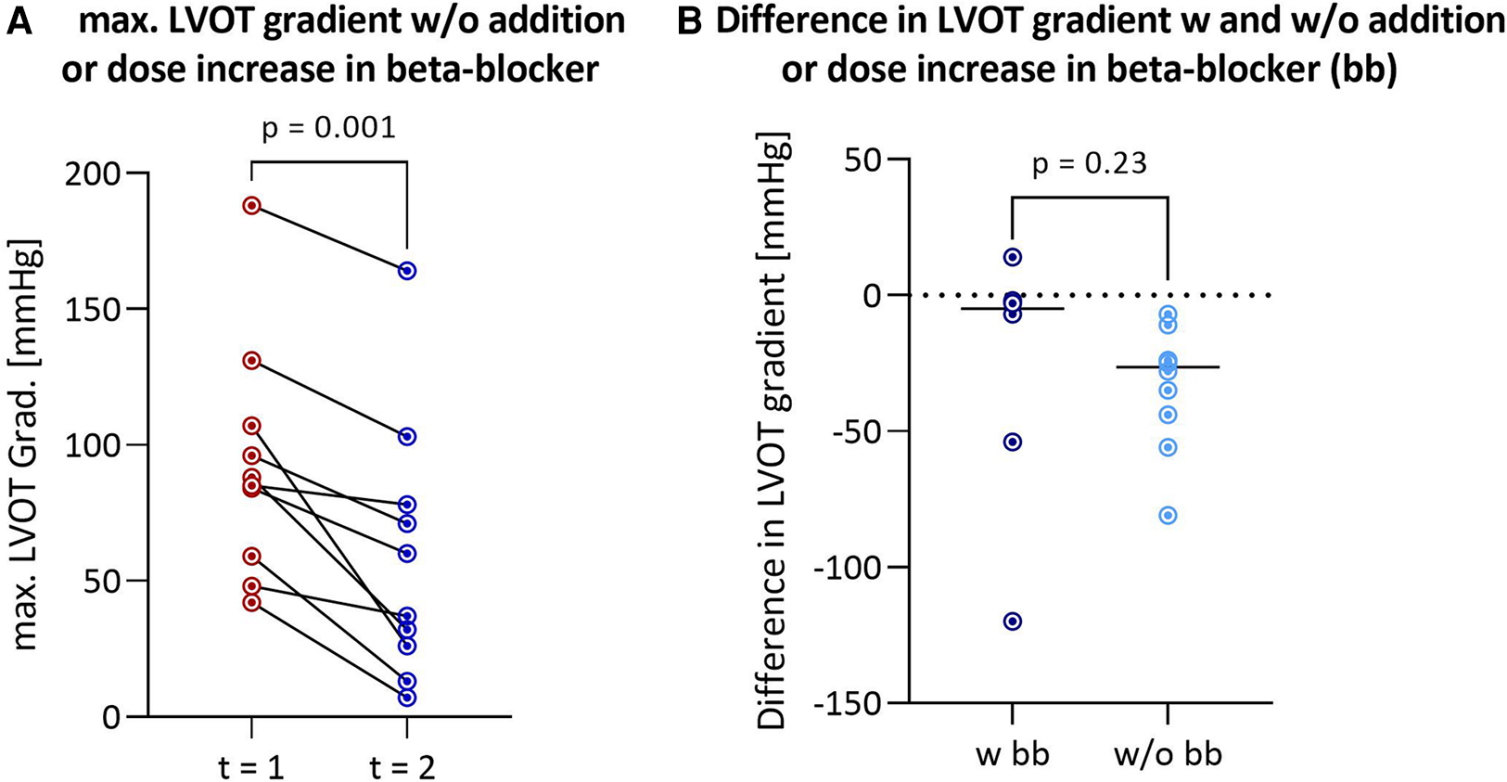
Reduction in LVOT gradient after discontinuation of afterload-reducing drugs in HOCM patients without addition or dose increase of a beta-blocker (*n* = 10) (**A**) comparison of differences in LVOT gradient between first and second outpatient visit between HOCM patients with and without addition or dose increase of a beta-blocker (**B**) LVOT, left ventricular outflow tract; HOCM, hypertrophic obstructive cardiomyopathy; bb, beta-blockers.

The median interval between the two outpatient visits was 193 [113–339] days in the cohort that discontinued afterload-reducing medication and 166 [47–365] days in the group that continued medication, and was not statistically significant (*p* = 0.52). Discontinuation of afterload-reducing medication resulted in a significant decrease in median LVOT gradient from 86.5 [60.5–109.3] mmHg to 61.5 [28.3–97.5] mmHg (*p* = 0.0004) (Table 1 and Figure 1A). This represents a mean LVOT gradient reduction of 31.7 ± 33.8 mmHg. Other echocardiographic parameters such as IVSd, LA PLAX, LVEDD, LV mass, LV mass-index, mitral valve regurgitation or diastolic dysfunction did not change significantly between outpatient visits. Similarly, symptoms such as dyspnea, assessed by the NYHA classification, syncope and palpitations did not change significantly in either group (Table 2). Table 2 shows that 100% of patients discontinued their ACEi therapy. One patient continued to take CCB-DHP but discontinued ACEis. There was also a clear but not significant increase in patients who started therapy with a mineralocorticoid receptor antagonist (MRA) (*p* = 0.12). Change in systolic (*p* = 0.12) and diastolic (*p* = 0.94) blood pressure did not differ between both patient groups. The subgroup analysis of patients who did not start or increase their beta-blocker dose at the same time is illustrated in Figure 2A. Again, a significant reduction in LVOT gradient was achieved (*p* = 0.001). A comparison between HOCM patients with and without addition or dose increase of a beta-blocker revealed that this had no significant impact on the change in the LVOT gradient (*p* = 0.23), as illustrated in Figure 2B.

Continuation of afterload-reducing medication resulted in a tendency towards a slight increase in median LVOT gradient from 56.5 [41.5–63.3] mmHg to 67.5 [46.5–79.8] mmHg by a mean of 14.2 ± 23.8 mmHg (*p* = 0.14) (Table 1 and Figure 1B). Other echocardiographic parameters such as IVSd, LAVI, LVEDD, mitral valve regurgitation or diastolic dysfunction and symptoms such as dyspnea, syncope or palpitations did not change significantly.

**Figure 3:**
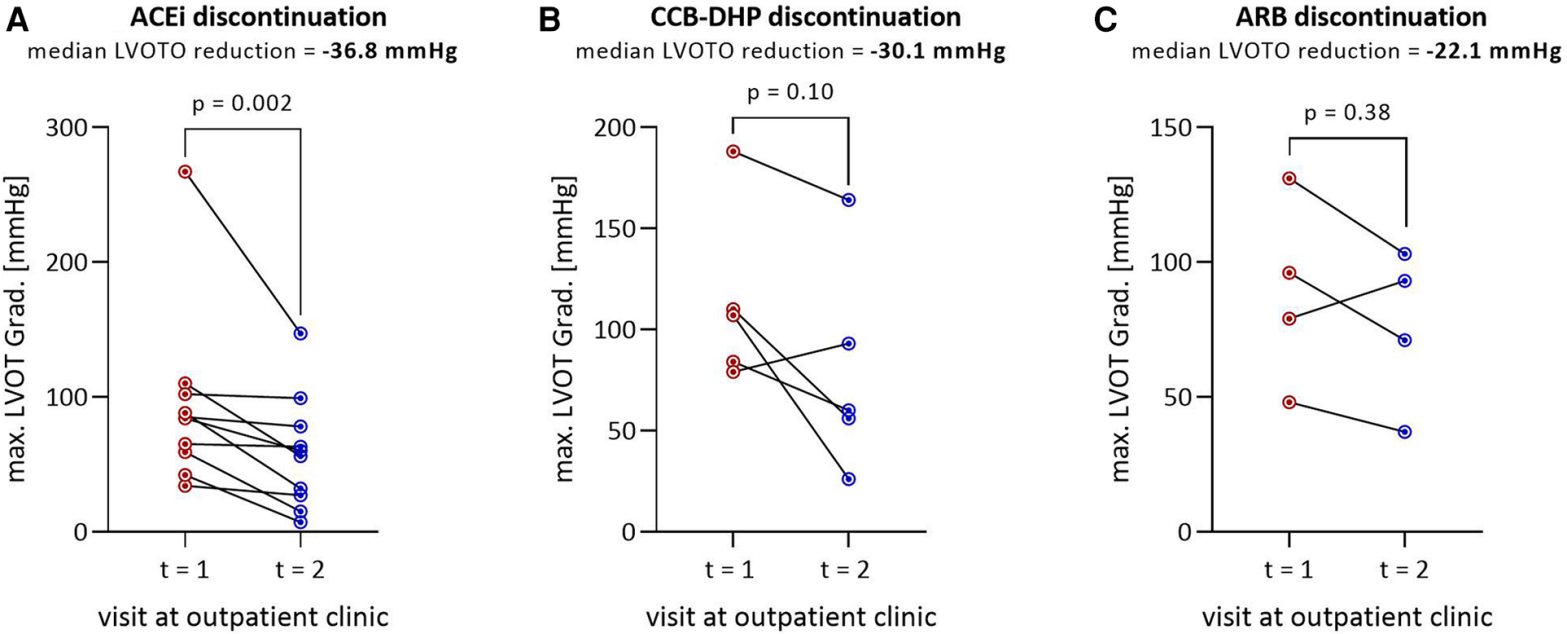
Reduction in LVOT gradient by class of discontinued drug: (**A**) ACEi discontinuation (*n* = 10), (**B**) CCB-DHP discontinuation (*n* = 5), (**C**) ARB discontinuation (*n* = 4). The median LVOT gradient reduction is shown below the title. The total number of patients in (**A–C**) is 19 instead of 16 because three patients discontinued ACEi and CCB-DHP or ARB and CCB-DHP simultaneously and therefore appear twice. in these figures. LVOT, Left ventricular outflow tract; LVOTO, left ventricular outflow tract obstruction; ACEi, angiotensin converting enzyme inhibitor; CCB-DHP, dihydropyridine calcium channel blocker; ARB, angiotensin-1 receptor blocker.

Figures 3A–C show the reduction in LVOT gradient by drug class: ACEis, CCB-DHPs and ARBs. Discontinuation of ACEis leads to a significant reduction in LVOT gradient, with a median reduction of −36.8 mmHg (Figure 3C). The LVOT gradient was reduced at follow-up in 3/4 of patients who stopped ARBs (median = −30.1 mmHg) and 4/5 of patients who stopped CCB-DHPs (median = −22.1 mmHg). Nevertheless, the reduction was not statistically significant probably due to the low number of patients.

Table 2 shows the changes between the first and second outpatient visits. For this purpose, the values of the second visit were subtracted from those of the first visit to present the differences. Apart from the change in medication and the associated reduction in the LVOT gradient (*p* = 0.0024), no parameters such as blood pressure, LVEDD, IVSd or mitral regurgitation developed significantly differently between the two patient groups. In the group of patients who discontinued the afterload-reducing drugs, one patient continued therapy with CCB-DHP (Table 2). This decision was made because this patient had previously been treated with an additional ACEi to manage AH. To prevent a potential extreme increase in blood pressure, only the ACEi was stopped at first and the medication was continued with a CCB-DHP.

## Discussion

The treatment of HOCM patients has garnered increased attention recently, mainly due to the introduction of a new class of drugs known as myosin inhibitors. The inaugural compound in this class—Mavacamten—has shown tremendous effects on LVOTO in two trials (19, 20). These results have prompted a recommendation for its use in the treatment of HOCM in the recently published guidelines on the treatment of cardiomopathies by the ESC (8). Nevertheless, treatment is recommended on top of therapy with beta-blockers or non-dihydropyridine-CCB after discontinuation of vasodilating drugs. However, there is limited data available on the direct effect of such discontinuation on the LVOTO. In this study, we aimed to investigate the impact of afterload-reducing drugs on the LVOT gradient and the potential benefits of discontinuing these medications.

Patients with HCM are inadvertently exposed to the administration of afterload-lowering agents for several reasons. Primarily, HCM often eludes accurate diagnosis because left ventricular hypertrophy is often mistakenly associated with persistent systemic AH. Moreover, individuals with HCM often have concomitant AH, a phenomenon described in a previous study with an incidence rate of 37% (21). The coincidence of these scenarios leads to the erroneous prescription of pharmacological agents known to reduce afterload, such as ACEis, ARBs, and CCB-DHPs. The ESC recommends several drugs for the treatment of AH and left ventricular hypertrophy as a consequence of long-term AH, including ACEis, ARBs, CCBs and diuretics (22). The most commonly used ones are ACEis and ARBs, both targeting the renin-angiotensin-aldosterone system (RAAS), a pivotal regulator of blood pressure. The secretion of the protease renin has an impact on various parameters, including renal perfusion pressure, sodium, and angiotensin II (23, 24). ACEis and ARBs interfere with this system either by inhibiting the formation of circulating angiotensin II or by blocking the angiotensin II subtype 1 (AT1) receptor, thereby reducing the stimulatory effects of angiotensin II on vasoconstriction, sodium/water retention, myocardial remodeling and left ventricular (LV) hypertrophy (25). Therefore, the desired effect is not only a reduction in arterial blood pressure but also the prevention of myocardial remodeling. ARBs and ACEis have repeatedly been shown to be effective in preventing LV remodeling in terms of a decrease in LV mass and an improved systolic myocardial velocity in several heart diseases (26, 27).

Previous studies investigating the potential beneficial effects of ARBs in HCM patients show conflicting results. In a study by Axelsson et al., losartan could not show a beneficial effect on cardiac function or exercise capacity (28). However, a smaller study by Penicka et al. demonstrated a significant reduction in left ventricular mass, improved LV function and exercise tolerance following treatment with candesartan (29). In addition, Shimada et al. observed a significant decrease in the extent of late gadolinium enhancement on MRI after treatment with losartan (30). Another study was also able to demonstrate that treatment with valsartan decreases type I collagen synthesis in HCM patients (31). However, these studies were conducted in HNCM patients and did not investigate the effects on left ventricular outflow tract obstruction.

The effect of ACEis has received noticeably less attention in previous studies compared to ARBs. Only one study has specifically examined the effects of ACEis in HCM patients. Combination therapy of intracoronary enalapril and sublingual captopril was administered, and the effects on the LVOT gradient, coronary blood flow and coronary flow reserve were investigated (32). The local application of enalapril into the coronary arteries during cardiac catheterization resulted in improved LVOT gradient, coronary blood flow and reserve. However, these beneficial effects were completely reversed by the subsequent systemic administration of captopril (32). These results are consistent with our observations, indicating that systemic administration of ACEis worsens outflow tract obstruction, leading to increased LVOT gradient.

CCB-DHP are another commonly prescribed class of drugs for the treatment of AH. The target structures are voltage-gated L-type calcium channels of vascular smooth muscle, directly affecting peripheral vascular resistance and arterial blood pressure by regulating calcium currents (33). There have been limited studies on the use of CCB-DHP in HCM patients. One study found that monotherapy with nifedipine did not increase the LVOT gradient (34). Nevertheless, combined with propranolol, the combination therapy was less effective than monotherapy with propranolol alone (35). The combination therapy even resulted in a worsening of symptoms. Clinically, CCB-DHP in HOCM patients should be avoided as far as possible.

ACEis and ARBs, as well as CCB-DHP cause vasodilation and, thus, a reduction in arterial blood pressure by reducing total peripheral resistance (36, 37). This mechanism poses a challenge for patients with HOCM since the obstruction increases pressure within the left ventricle and decreases it behind the aortic valve and therefore can exacerbate the LVOT gradient. Increasing the gradient can worsen symptoms such as dyspnoea or dizziness and lead to syncope. Therefore, current guidelines do not recommend these three classes of drugs for HOCM (6, 7).

According to the mechanism of action described above, our results confirmed that discontinuing these drug classes decreases the gradient. The mean decrease in the LVOT gradient was 31.7 ± 33.8 mmHg in patients who discontinued afterload-reducing drugs. The observed reduction in the LVOT gradient after discontinuation was primarily seen by the discontinuation of ACEis, both in terms of numerical differences and magnitude of effect. LVOT gradient reduction occurred in 75% and 80% of cases in the ARB and the CCB-DHP group, respectively. No significant reduction in LVOT gradient was observed in those subgroups (CCB-DHP, ARB), although this may be attributed to the small number of cases. In clinical application, afterload-lowering therapy with ACEis, ARBs and CCB-DHPs should be avoided in most cases of patients with HOCM.

In some patients, beta-blocker therapy, known to have an anti-obstructive effect, was initiated or increased concurrently with the discontinuation of afterload-reducing medication. However, the reduction in gradient remained significant even in the subgroup where neither beta-blocker therapy was started, nor the dose was increased (Figure 2A).

In our study, although the LVOT gradient was reduced after switching medication, significant differences in symptomatology did not result statistically, especially concerning dyspnea and the associated categorizable variable NYHA classification. This is most likely due to the small number of patients. Although the changes in arterial blood pressure are statistically insignificant, a slight non-statistically significant increase in blood pressure was observed in patients who stopped taking the afterload-lowering drugs. Therefore, blood pressure should be monitored regularly, and medications such as verapamil or beta-blockers should be used, or their dose should be increased to maintain blood pressure in the desired range.

Mechanistically, the reduction in left ventricular outflow tract obstruction (LVOTO) observed in our study could significantly impact cardiac function. It has been suggested that alleviating LVOTO could lower wall tension, thereby reducing myocardial oxygen consumption and positively affecting left atrial pressure and remodeling (38, 39). These physiological changes may improve the metabolic profile and overall cardiac function in hypertrophic cardiomyopathy (HCM) patients and associated obstructive physiology.

A primary limitation of this study is the small number of cases. This is because patients are advised to discontinue afterload-lowering therapy even before further assessment in our dedicated outpatient clinic, often at our request, especially if the reported symptoms are pronounced.

## Conclusion

Despite the relatively small number of patients included in the study, the results show that simple changes in therapy can significantly reduce LVOTO in patients with HOCM. As a clinically relevant conclusion, the first therapeutic step is discontinuing afterload-lowering drugs and the guideline-based initiation of beta-blockers or non-DHP CCB.

## Data Availability

The raw data supporting the conclusions of this article will be made available by the authors, without undue reservation.
