# Supplementary material for: Discontinuation of afterload-reducing drugs decreases left ventricular outflow tract obstruction in hypertrophic obstructive cardiomyopathy
Source: Front Cardiovasc Med. 2024 Jul 16;11:1403422. doi: 10.3389/fcvm.2024.1403422 (PMC11286422; doi:10.3389/fcvm.2024.1403422)
Supplement: Supplementary file 1 [file Table1.docx]

Supplementary Material

Discontinuation of afterload-reducing drugs decreases left ventricular outflow tract obstruction in hypertrophic obstructive cardiomyopathy

**Anselm A. Derda^1,2,*^, Malin Abelmann^1,2,*^, Kristina Sonnenschein^1,2^, Jan-Thorben Sieweke^1^, Udo Bavendiek^1^, Johann Bauersachs^1^, Thomas Thum^2,†^, Dominik Berliner^1,†,#^.**

**^1^Department of Cardiology and Angiology, Hannover Medical School, Hannover, Germany.**

**^2^Institute of Molecular and Translational Therapeutic Strategies (IMTTS), Hannover Medical School, Hannover, Germany.**

*** These authors contributed equally to this work and share first authorship.**

**^†^ These authors contributed equally to this work and share last authorship.**

**#Corresponding author:
Dr. Dominik Berliner**

**Hannover Medical School**

**Carl-Neuberg-Str. 1**

**30625 Hannover, Germany**

**Email:** [**Berliner.dominik@mh-hannover.de**](mailto:Berliner.dominik@mh-hannover.de)

**Phone: 0511 532 2226**

**Supplementary Table 1:**

**Baseline characteristics of HOCM patients who discontinued afterload-reducing medication and HOCM patients who continued afterload-reducing medication at their first outpatient visit at MHH, subdivided by sex.**

|  | First visit  (Discontinuation)  n = 16 | | First visit  (Continuation)  n = 8 | |
| --- | --- | --- | --- | --- |
|  | male  n = 7 | female  n = 9 | male  n = 4 | female  n = 4 |
| Demographics |  |  |  |  |
| Age (years) | 65.9 ± 9.8 | 69.6 ± 4.4 | 62.0 ± 7.9 | 63.6 ± 7.7 |
| Systolic blood pressure (mmHg) | 143.1 ± 20.7 | 128.9 ± 15.8 | 141.8 ± 15.9 | 127.0 ± 8.2 |
| Diastolic blood pressure (mmHg) | 73.4 ± 6.4 | 73.0 ± 12.1 | 80.3 ± 6.8 | 75.0 ± 9.5 |
| Arterial hypertension (%) | 7 (100) | 9 (100) | 4 (100) | 4 (100) |
| Pharmacotherapy |  |  |  |  |
| Beta-blocker (%) | 4 (57.1) | 6 (66.7) | 2 (50.0) | 3 (50.0) |
| ACEi (%) | 5 (71.4) | 5 (55.6) | 3 (75.0) | 4 (100) |
| ARB (%) | 2 (28.6) | 2 (22.2) | 0 (0) | 0 (0) |
| CCB-DHP (%) | 2 (28.6) | 4 (44.4) | 1 (25.0) | 1 (25.0) |
| Non-DHP CCB (%) | 2 (28.6) | 0 (0) | 1 (25.0) | 0 (0) |
| MRA (%) | 0 (0) | 2 (22.2) | 0 (0) | 0 (0) |
| Diuretics (%) | 3 (42.9) | 6 (66.7) | 3 (75.0) | 1 (25.0) |
| Symptoms |  |  |  |  |
| NYHA I or II | 6 (85.7) | 6 (66.7) | 3 (75.0) | 3 (75.0) |
| NYHA III or IV | 1 (14.3) | 3 (33.3) | 1 (25.0) | 1 (25.0) |
| Palpitations (%) | 1 (14.3) | 2 (22.2) | 0 (0) | 1 (25) |
| Syncope (%) | 0 (0) | 0 (0) | 0 (0) | 0 (0) |
| Echocardiography |  |  |  |  |
| LVOT gradient max. (mmHg) | 85.0  [59.0-96.0] | 102.0  [60.5-159.5] | 54.0  [41.5-82.8] | 58.0  [38.5-63.3] |
| IVSd (mm) | 17.3 ± 2.3 | 20 ± 2.3 | 21.5 ± 1.7 | 16.5 ± 3.1 |
| LA PLAX (mm) | 47.4 ± 3.6 | 44.1 ± 6.9 | 40.0 ± 5.5 | 38.5 ± 6.2 |
| LVEDD (mm) | 40.0 ± 11.5 | 42.2 ± 4.1 | 45.0 ± 7.8 | 34.3 ± 8.3 |
| Diastolic dysfunction (%) | 7 (100) | 9 (100) | 4 (100) | 4 (100) |
| Mitral valve regurgitation at least moderately severe (%) | 4 (57.1) | 6 (66.7) | 1 (25.0) | 1 (25.0) |
| LVEF ≥ 50% | 7 (100) | 9 (100) | 4 (100) | 4 (100) |

HOCM, Hypertrophic obstructive cardiomyopathy; ACEi, Angiotensin converting enzyme inhibitor; ARB, Angiotensin-1 receptor blocker; CCB-DHP, Dihydropyridine calcium channel blocker; non-DHP CCB, non-dihydropyridine calcium channel blocker; MRA, Mineralocorticoid receptor antagonist; NYHA, New York Heart Association; LVOT, Left ventricular outflow tract; IVSd, Interventricular septal thickness in diastole; LA PLAX, Left atrial parasternal long axis; LVEDD, Left ventricular end diastolic diameter; LVEF, Left ventricular ejection fraction.
